# A systematic investigation of production of synthetic prions from recombinant prion protein

**DOI:** 10.1098/rsob.150165

**Published:** 2015-12-02

**Authors:** Christian Schmidt, Jeremie Fizet, Francesca Properzi, Mark Batchelor, Malin K. Sandberg, Julie A. Edgeworth, Louise Afran, Sammy Ho, Anjna Badhan, Steffi Klier, Jacqueline M. Linehan, Sebastian Brandner, Laszlo L. P. Hosszu, M. Howard Tattum, Parmjit Jat, Anthony R. Clarke, Peter C. Klöhn, Jonathan D. F. Wadsworth, Graham S. Jackson, John Collinge

**Affiliations:** MRC Prion Unit and Department of Neurodegenerative Disease, UCL Institute of Neurology, National Hospital for Neurology and Neurosurgery, Queen Square, London WC1N 3BG, UK

**Keywords:** prion, prion disease, prion protein, prion amyloid, synthetic prions

## Abstract

According to the protein-only hypothesis, infectious mammalian prions, which exist as distinct strains with discrete biological properties, consist of multichain assemblies of misfolded cellular prion protein (PrP). A critical test would be to produce prion strains synthetically from defined components. Crucially, high-titre ‘synthetic' prions could then be used to determine the structural basis of infectivity and strain diversity at the atomic level. While there have been multiple reports of production of prions from bacterially expressed recombinant PrP using various methods, systematic production of high-titre material in a form suitable for structural analysis remains a key goal. Here, we report a novel high-throughput strategy for exploring a matrix of conditions, additives and potential cofactors that might generate high-titre prions from recombinant mouse PrP, with screening for infectivity using a sensitive automated cell-based bioassay. Overall, approximately 20 000 unique conditions were examined. While some resulted in apparently infected cell cultures, this was transient and not reproducible. We also adapted published methods that reported production of synthetic prions from recombinant hamster PrP, but again did not find evidence of significant infectious titre when using recombinant mouse PrP as substrate. Collectively, our findings are consistent with the formation of prion infectivity from recombinant mouse PrP being a rare stochastic event and we conclude that systematic generation of prions from recombinant PrP may only become possible once the detailed structure of authentic *ex vivo* prions is solved.

## 1. Introduction

Prions are infectious agents responsible for the transmissible spongiform encephalopathies or prion diseases, lethal neurodegenerative conditions including Creutzfeldt–Jakob disease in humans, scrapie in sheep and goats, bovine spongiform encephalopathy in cattle, and chronic wasting disease in deer and elk [1,2]. Prions are thought to consist of fibrillar polymers of misfolded cellular prion protein (PrP^C^) that propagate by recruitment of host PrP^C^ leading to elongation and fission [3]. It is increasingly thought that prion-like processes, with the spread of propagating proteopathic seeds, underlie the pathogenesis of more common neurodegenerative diseases, such as Alzheimer's and Parkinson's, leading to a wider interest in understanding prion structure and strain diversity [4]. Historically, it has proved difficult to adequately purify *ex vivo* prions and obtain sufficiently homogeneous material for high-resolution structural analysis; hence the structure of infectious prions and the structural basis of prion strain diversity remain unresolved. The ability to make synthetic prions, at will, from purified recombinant PrP (recPrP) would not only establish the protein-only hypothesis beyond doubt, but would also provide a model system where the assembly mechanism and structural properties of the infectious agent could be elucidated. Were relatively homogeneous prions produced from recPrP, it would be expected that they could be diluted many million-fold and still result in 100% lethality in susceptible animals [3]. In the absence of such results, structural studies would be futile (and indeed would report on the large excess of uninfectious material [3]).

Early reports of structural conversion of the predominantly *α*-helical PrP^C^ conformation to isoforms with properties in common with disease-associated forms (PrP^Sc^) isolated from infected tissue [5–9] were not accompanied by evidence of infectivity. However, there have since been multiple reports of *de novo* production of prions from bacterially expressed recPrP using a range of methods [10–14]. Interpretation of some of these reports is complicated either by bioassay using transgenic mice with high levels of overexpression of PrP (which develop spontaneous neurodegeneration) or by use of *in vitro* prion amplification methods such as protein misfolding cyclic amplification (PMCA), which are capable of amplifying a single prion particle to a concentration that can be detected in rodent bioassay, leading to concerns of contamination or amplification of naturally occurring prions that might form spontaneously in mammalian brain with a low stochastic frequency [15]. Importantly, however, none of these studies have reported a systematic method for production of large quantities of high-titre material that would be suitable for detailed structural analyses.

In our own earlier work, while we did occasionally observe neurological disease in rodents challenged intracerebrally with various preparations of recPrP which could be passaged, we were unable to do this reproducibly and concluded that if infectivity were produced in these experiments at all, it was of extremely low titre and useless for structural characterization. Optimization of prion synthesis via lengthy rodent bioassay massively restricts the range of conditions that can be tested, and for this reason we established a novel strategy to allow rapid exploration of a large array of conditions, additives and potential cofactors in a manner conceptually similar to a matrix approach to protein crystallization and rapidly screening for infectivity using a cell-based prion bioassay, the scrapie cell assay (SCA) [16], which we have automated (ASCA) and used for high-throughput prion determination [17,18]. It would then be possible to focus on a subset that could be iteratively optimized to produce high-titre prions that could be investigated further. A further advantage of using a cell-based system to detect synthetic prions is that nascent prions may be particularly labile and sensitive to *in vivo* clearance and other defence mechanisms. Conversely, it might be argued that the ASCA is limited to detection of RML or 22 L-like prions [16]. However, prion strains appear to constitute a molecular ensemble or quasi-species in dynamic equilibrium [3,19]. The population of different strains must follow statistical thermodynamic distributions with varying energy barriers between them. Even where the barriers may be high and rates of inter-conversion slow, there is clear evidence for the coexistence of several conformations which can evolve under selection [20] such that PK1 cells may select RML-like prions via conformational selection [3,21]. That the RML prion strain can be derived by single passage of cattle BSE prions in SJL inbred mice also suggested RML may be a particularly preferred pathogenic PrP conformer in mice [22]. Notably, synthetic prions generated by Ma and co-workers [14] could infect cells in culture but produced a non-RML prion strain phenotype when subsequently inoculated in mice. These findings support the ability of cell culture infectivity assays to detect infectious prions with novel strain properties.

## 2. Results

**Figure 1.**
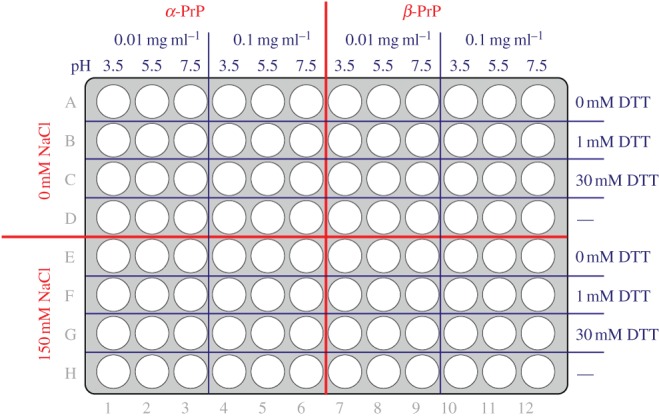
Plate format for matrix core conditions. Master 96-well plates were prepared so that wells contained *α*- or *β*-PrP at a final protein concentration of 0.01 or 0.1 mg ml^−1^ in a fixed set of core solvent conditions of defined pH and concentration of DTT and NaCl. Condition variables and additives (table 1) were introduced to this master plate format, after which plates were incubated for different time periods at different temperatures (as detailed in Methods) prior to infectivity measurement in the ASCA.

**Figure 2.**
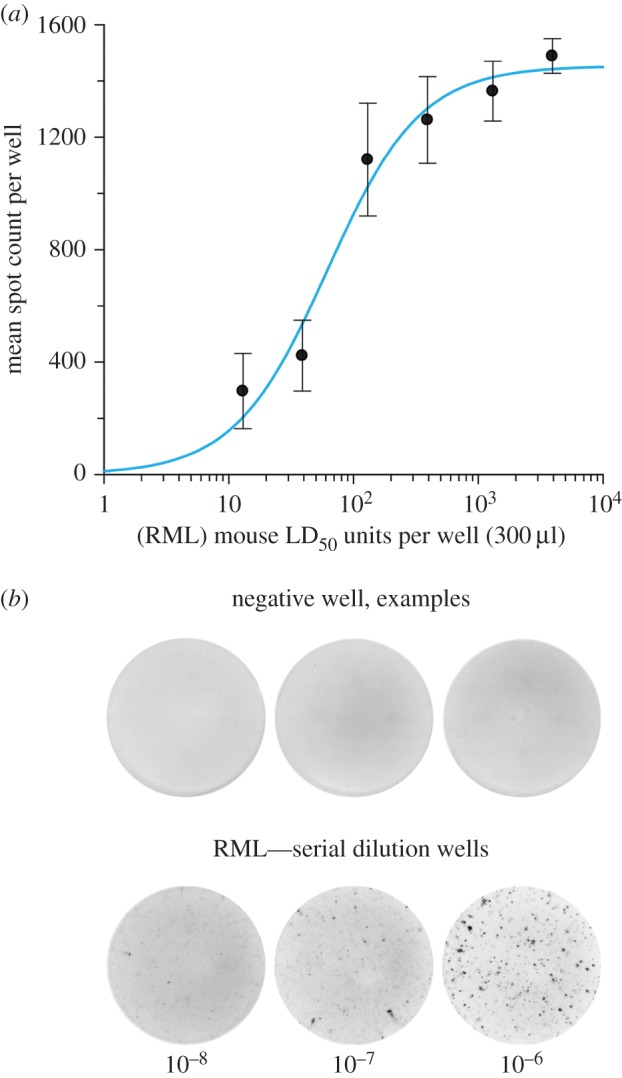
ASCA measurement of RML prion infectivity. (*a*) Dose–response curve of RML prion infection of tissue culture wells containing 18 000 PK1/2 cells. The number of mouse intracerebral LD_50_ units of RML prions applied per well is plotted against mean spot number per well ± s.e.m. (*n* = 6) determined at the fourth cell split. (*b*) Representative examples of positive and negative ELISPOT wells.

Prior to design of the matrix of conditions to be explored, a comprehensive characterization of the biochemical properties and physical stability of *ex vivo* RML prions was performed (data not shown). Additional conditions were added in response to published data on putative cofactors [11,12,14,23,24]. We conducted an unprecedented search for conditions that could produce high titres of synthetic infectivity in a reproducible manner with defined solvent and environmental conditions. To achieve this, recPrP folded into a native alpha-helical conformation (*α*-PrP) of a beta-sheet-enriched conformation (*β*-PrP) [5] was exposed to differing solvent conditions varying with respect to pH, redox potential and ionic strength. The variables chosen were designed to manipulate the making and breaking of disulfide bonds, the net protein charge, the strength of electrostatic and non-polar interactions, and the rate of protein–protein collisions. These variable solvent conditions were replicated with extending incubation times of 1 h, overnight and three months, with further nested variation of temperature comprising room temperature, 4, 37 and 55°C. These ‘core’ conditions were explored on 96-well plates (figure 1) with the incorporation of condition variables and additives (table 1), based upon previous reports in the literature or rational consideration of what may promote ordered aggregation events and relevant cofactors. The secondary variables included proteolysis (to enrich the proportion of resistant material), brain homogenate (to supply cofactors), phospholipids/detergents, scaffolding factors (e.g. heparin) and metals that may affect the folded conformation. We specifically explored the effects of reaction surfaces, notably stainless steel, given previous findings that prions can be efficiently concentrated on steel wires, allowing greatly enhanced prion detection [25], and may form spontaneously on metal surfaces [15]. We also investigated fibrils generated from recPrP by adapting published methods which reported infectivity [10,26]. Prion infectivity was assayed using a modification of the ASCA. A serial dilution of a titred RML prion homogenate was assayed in parallel as a positive control and for quantification of any potential synthetic prions (figure 2). Samples were considered positive on the basis of three criteria: (i) the presence of a high number of PrP^Sc^-positive cells (defined by protease-resistant PrP deposits or ‘spot’ counts; see the electronic supplementary material, Methods; >background mean + 10 s.d.), (ii) evidence of prion propagation (increasing number of positive cells over two successive splits) and (iii) the reproducibility of duplicates.

**Table 1.** Condition variables and additives applied to the core matrix.

variable condition	details^a^
limiting proteolysis	0.1 µg ml^−1^ proteinase K, 37°C, 30 min 1.0 µg ml^−1^ thermolysin, 37°C, 30 min
reaction surface	discs (surgical steel, AISI 304, 316) wires (surgical steel, AISI 304, 316) polypropylene, polycarbonate, glass, PTFE

additives	details^a^
Pro-ject lipids	commercial transfection reagent anionic/cationic mixes
silk, heparin, glycogen, ferritin	protein scaffolds
surfactants	0.1 and 5.0% (w/v) SDS 0.1 and 1.0% (v/v) Triton X-100 0.1 and 1.0% (v/v) Tween-20
metal ions	Cu^2^^+^ at 1 : 1 and 5 : 1 molar ratio to PrP Zn^2+^ at 1 : 1 and 5 : 1 molar ratio to PrP Mn^2+^ at 1 : 1 and 5 : 1 molar ratio to PrP
brain or cell homogenate	1.0% (w/v) normal mouse brain PMCA substrate normal mouse brain PK1 cell membrane fraction GuHCl denatured RML-infected mouse brain
previously reported	PolyAdenosine [11] 1-palmitoyl-2-oleoylphosphatidylglycerol [14] Phosphotidylethanolamine [24]
fibrils	recPrP fibrils generated from RML prion-seeded polymerization reactions

^a^Full details can be supplied on request.

Positive and negative controls were crucial for quality control and their values had to fall within a validated range (non-infected cells producing a background below a defined threshold; see Methods) or the experiment was repeated. If any positives were reproducible, the pooled conditioned medium produced by the apparently infected cells was assayed by SCA. In parallel, PrP-silenced PK1 cells, whose *Prnp* expression was silenced by two shRNAs directed against the 3′-UTR and which are refractory to prion infection [27] (electronic supplementary material, figure S1), were infected with the same conditions to exclude the possibility of false positives resulting from recPrP aggregation. If the repetition experiment was successful, the formation of putative synthetic prions was assessed by standard mouse bioassay using wild-type FVB/N mice. In total, we screened 19 468 unique conditions (approx. 25 000 including repeated conditions).

**Figure 3.**
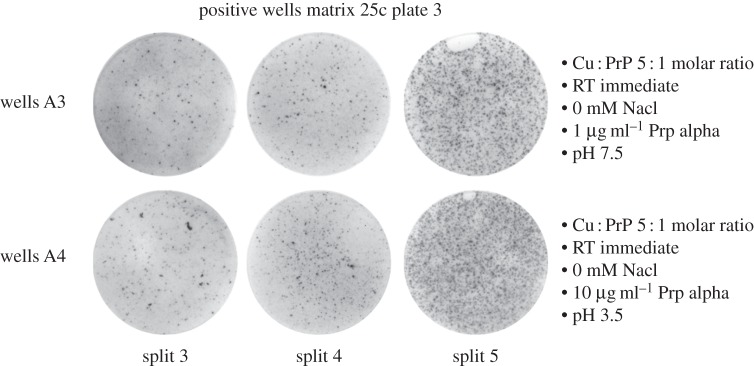
Example positive wells from the matrix. ELISPOT wells containing spots with the characteristic visual appearance seen in prion bioassay by SCA were observed to increase successively over cell splits three to five. The matrix conditions that generated these positive findings are detailed next to the wells and were observed in the presence of copper ions at a 5 : 1 molar ratio with PrP and incubation at room temperature (RT) for 1 h (immediate) prior to inoculating the cells. These findings were not consistently reproducible.

Positive wells were recorded using our stringent criteria from 480 unique conditions (electronic supplementary material, table S1). These positive wells had the characteristic visual appearance seen in our extensive experience of prion bioassay by SCA; indeed, they were indistinguishable from RML prion-infected positive control wells (figure 3). However, despite multiple attempts, we were not able to serially propagate such isolates in PK1 cells. Furthermore, the conditions were not reliably reproducible to enable large-scale repeats to harvest cells for conventional mouse bioassay.

In addition to systematic screening of this matrix of conditions, we also adapted specific published methods reported as producing synthetic prions [10,12–14], including those that relied on initial seeding with authentic *ex vivo* prions. In common with others [28], we did not find evidence of significant titre in the materials generated by these methods (either by ASCA or by conventional mouse bioassay; electronic supplementary material, table S2). To date the prion-seeded reaction products of recombinant mouse PrP generated by Supattapone and colleagues [29] represents the only preparation with a specific infectivity that might be compatible with meaningful structural analyses. However, the prion seed used to initiate these reactions has only been generated by these researchers to date [14,29,30] and our own attempt to generate such a prion seed by adapting their published methods [14] was unsuccessful (electronic supplementary material, table S2). In this regard, the method has been reported by the authors to not always lead to the formation of infectious prions [30].

Our own observations of occasional positive wells in ASCA and the sporadic reports of synthetic infectivity in the literature are consistent with the formation of infectivity being a rare stochastic event which is difficult to reproduce *in vitro*. Under our matrix conditions, the ASCA is able to robustly detect prions at a concentration of 10 LD_50_ units per 300 µl assay well (figure 2). For the highest concentrations of recPrP analysed (100 µg ml^−1^ reactions), each ASCA well contained 2.67 µg of PrP, which is equivalent to the PrP content of 50 mg of RML prion-infected mouse brain that contains approximately 10^8.1^ intracerebral LD_50_ units. As the ASCA robustly detects as little as 10 LD_50_ units per well our assay sensitivity has the ability to detect prions at 1 part in more than 10 million of total PrP by weight. The rare events that led to positive wells produced at best minimal titres of infectivity and the inability to reproduce positivity in such conditions precluded iterative optimization of conditions in the hope of progressively increasing titre towards the aim of generating material suitable for structural study.

## 3. Discussion

Our findings, systematically screening unprecedented numbers of conditions, highlight the difficulty of producing synthetic prion infectivity from bacterially expressed recPrP. It is possible that efficient prion production requires eukaryotically expressed, post-translationally modified PrP and/or specific cofactors. Additional factors may also include specific biological replication sites or surfaces. Highly specific quaternary structural features may be required in PrP polymers to render them efficient *in vivo* pathogens that would form at extremely low probability *in vitro*. While it is possible that such rare prion assemblies would then be amplified in a biological system, as may have occurred transiently in the SCA and robustly in some published *in vivo* studies, this of course then leads to the same problem of purification prior to structural analysis that has limited progress with conventional prions.

One important caveat of our screening approach for synthetic prions is that we are necessarily limited to detecting prion strains with replication rates that not only exceed their rates of degradation but also exceed the rate at which PK1 cells divide [16]. Increasing the number of cell passages beyond the standard SCA format would not therefore improve the assay's sensitivity or its ability to detect a greater range of possible synthetic prion strains. Instead alternative cell types that have different rates of division in culture might be required in order to detect slowly replicating synthetic prion strains [31]. However, repeating the screening that we describe with different cell types would be a highly challenging undertaking. A more promising approach may come from progress with purification and structural characterization of *ex vivo* prions [32], and in understanding cofactors present in normal brain tissue necessary for efficient *in vitro* prion amplification. These data can then be used to guide more focused approaches to eventually produce homogeneous synthetic prions that would allow atomic resolution structure determination.

## 4. Material and methods

### 4.1. Research governance

Work with prion-infected samples was conducted in microbiological containment level 3 facilities with strict adherence to safety protocols.

### 4.2. Preparation of recombinant prion protein master plates

Recombinant mouse PrP (*Prnp* allele a, residues 23–231) was prepared and folded in a native alpha-helical conformation (*α*-PrP) or a beta-sheet-enriched conformation (*β*-PrP) using buffer conditions described previously [33]. Full details can be supplied on request. Concentrated solutions of *α*- or *β*-PrP were dialysed into 10 mM bis-tris buffer (pH 6.5) or 10 mM sodium acetate buffer (pH 4.0), respectively, and then adjusted with the cognate buffer to give a final protein concentration of 1 mg ml^−1^. Immediately prior to use 1 mg ml^−1^ solutions were diluted to 0.02 or 0.2 mg ml^−1^ protein with 10 mM bis-tris/10 mM sodium acetate buffer adjusted to pH 3.5, pH 5.5 or pH 7.5. Master plates (Corning 96-well flat bottom microplates; Fisher Product Code: 10687551) were prepared so that wells contained *α* or *β* PrP at a final protein concentration of 0.01 or 0.1 mg ml^−1^ in a fixed set of core solvent conditions (figure 1) in the presence or the absence of variable additives (table 1) and a total final reaction volume of 80 µl. This was accomplished by adding 40 µl of 0.02 or 0.2 mg ml^−1^ *α*- or *β*-PrP to each well followed by appropriate additions of salt (2.4 µl of 5 M NaCl in water, to give 150 mM final concentration), DTT (2.5 µl of 32 or 960 mM DTT prepared in water, to give 1 or 30 mM final concentration) and additives (variable volumes), after which water was added to give a final reaction volume of 80 µl. Master plates were sealed and incubated without agitation for 1 h (immediate) or 16 h (overnight) at varying temperatures (4°C, room temperature, 37°C and 55°C). Some of the reactions were also examined after three-month incubation at 4°C or room temperature. At the end of the incubation, plates were unsealed and 220 µl tissue culture medium (OFCS; see below) added to each well and thoroughly mixed using a Biomek FX liquid handling robot to give a final volume of 300 µl. A total of 100 µl aliquots from each well were then directly applied to prion-susceptible cells (see below).

### 4.3. Automated scrapie cell assay

Prion-susceptible PK1/2 cells (a line derived from N2a cells that are highly susceptible to RML prions [16]) were routinely grown in OFCS medium (Opti-MEM, containing 10% fetal calf serum; 100 U ml^−1^ penicillin and 100 µg ml^−1^ streptomycin; Invitrogen, UK) using 15 cm Petri dishes. Harvested cells were manually dispensed into 96-well plates (Costar flat bottom 96-well plates; Corning, UK) at a density of 18 000 cells per well 24 h prior to infection and kept at 37°C in a 5% CO_2_ incubator. The following day the cell medium was removed and replaced with 200 µl fresh OFCS, after which 100 µl of the reaction solution from the PrP master plates (see above) was applied using a Biomek FX liquid handling robot. To verify the susceptibility of each batch of PK1/2 cells to prion propagation, an additional plate of cells was treated with 10% (w/v) RML-infected mouse brain homogenate (serially diluted threefold in OFCS in the range 3 × 10^−5^ to 1 × 10^−7^) and processed in parallel. After 3 days of infection, cells were resuspended using the Biomek FX, after which 37.5 µl of cell suspension from each well was transferred into a new 96-well plate containing 262.5 µl OFCS (1 : 8 split) and cells grown to confluence for 3 days. After two further 1 : 8 splits (as described above), an aliquot of 85 µl of cell suspension from each well (containing approx. 25 000 cells) was transferred into ELISPOT (Multi Screen Immobilon-P, Millipore, UK) plates while a second aliquot of 37.5 µl cell suspension was transferred to new a split plate containing 262.5 µl OFCS. This procedure was repeated for two further 1 : 8 splits. At the time of their collection, ELISPOT plates were vacuum drained and dried at 50°C prior to storage at 4°C until further processing.

### 4.4. ELISPOT plate development

Stored ELISPOT plates were warmed to room temperature, after which 60 µl of 1 µg ml^−1^ proteinase K (PK) (Roche, UK) in lysis buffer (50 mM Tris HCl, pH 8 containing 150 mM NaCl, 0.5% (w/v) sodium deoxycholate and 0.5% (v/v) Triton-X 100) was added to each well and incubated for 60 min at 40°C. The plates were washed (2 × 160 µl PBS) after which 120 µl of 3 M guanidinium thiocyanate prepared in 10 mM Tris HCl pH 8.0 was added to each well for 20 min. The wells were washed (7 × 160 µl PBS) and 150 µl of Superblock dry blend blocking buffer (Perbio, UK) was added to each well and incubated for 1 h. Following vacuum removal of Superblock each well was incubated with 0.55 µg ml^−1^ anti-PrP monoclonal antibody ICSM18 (D-Gen Ltd, London) prepared in TBST (10 mM Tris HCl, pH 8, 150 mM NaCl, 0.1% (v/v) Tween-20) containing 1% (w/v) non-fat dry milk for 1 h. After washing (5 × 160 µl TBST), wells were incubated for 1 h with 60 µl goat anti-mouse alkaline phosphatase-conjugated anti-IgG1 (Southern Biotechnology Associates, USA) diluted 1 : 8000 in TBST containing 1% (w/v) non-fat dry milk. Following washing (5 × 160 µl TBST), wells were incubated for 35 min with 50 µl AP dye (Bio-Rad, USA). The plates were then washed twice with water, dried and stored at −20°C. Spot counts (reporting PK-resistant PrP-positive cells; see below) were determined with a Zeiss KS ELISPOT system (Stemi 2000-C stereo microscope equipped with a Hitachi HV-C20A color camera, a KL 1500 LCD scanner and Wellscan software from Imaging Associates, Oxfordshire, UK) as described previously [16] or more recently using a Bioreader 5000-Eß system (BioSys, Karben, Germany).

### 4.5. Quantifying prion titre

ELISA detection of protease-resistant PrP on prion-infected cells on ELISPOT plates results in the appearance of focal PrP deposit ‘spots' that have a morphological appearance distinct from any non-specific background immuno-reactivity seen on the plate. A spot identifies when a prion-infected cell and spot count within a defined population of cells is proportional to the prion titre of the sample used to infect the cells, with spot counts rising (as prions propagate) over successive cell splits [16]. The dose response is dynamic between approximately 50 and 1000 spots per well; however, because the assay is nonlinear, every experiment must include concomitant assay of a serial dilution of RML prions of known prion titre (intracerebral LD_50_ units/ml determined from rodent bioassay) to produce a standard curve that unknown samples can be calibrated against. Two stock pools of 10% (w/v) RML brain homogenate (designated I6200 and I8700) were used as reference preparations and showed comparable prion infectivity titres in the scrapie cell end point assay [16] of approximately 10^6.5^ tissue culture infectious units (TCIU) ml^−1^ in PK1/11 cells [32,34,35] or 10^7.7^ TCIU ml^−1^ in PK1/2 cells [18]. I6200 reported a prion titre of 10^7.3^^±^^0.5^ (mean ± s.d.) intracerebral LD_50_ units ml^−1^ when endpoint titrated six times in Tg20 mice [32] that overexpress mouse PrP on a *Prnp^o/o^* background [36] and I8700 reported a prion titre of 10^7.2^ intracerebral LD_50_ units ml^−1^ when endpoint titrated once in Tg20 mice [32]. Figure 2 exemplifies a standard curve generated by the ASCA, showing robust detection of spots after infecting PK1/2 cells with 10 intracerebral LD_50_ units of RML prions per well. Matrix samples were scored positive on the basis of three criteria: (i) significant spot counts (>background mean + 10 standard deviations), (ii) evidence of prion propagation (increasing spot counts over two successive cell splits measured between splits three and five) and (iii) the reproducibility of duplicates for each sample.

### 4.6. Silencing of *Prnp* expression in susceptible N2a cells

To silence *Prnp* expression in susceptible PK1 cells, the 19mer TAGGAGATCTTGACTCTGA was cloned into the vector pSUPER.retro.puro (Oligoengine). Sense and antisense target sequences, flanked by a hairpin, TTCAAGAGA, were inserted at the BglII and HindIII sites of pSUPER.retro.puro essentially as described previously [27].

### 4.7. Adaptation of previous methods reporting synthetic prion generation

Recombinant murine PrP encompassing the complete amino acid sequence of the mature protein from residues 23 to 231 (PrP^23–231^) of *Prnp* allele a, or a truncated form representative of the structure C-terminal domain PrP^91–231^, were subjected to a modification of conditions previously described to generate synthetic prions. In the case of material produced by Legname *et al.* [10], the construct PrP^91–231^ was used and fibrillized at 0.5 mg ml^−1^ in 20 mM sodium acetate (pH 5.0), 1 M GuHCl, 3 M urea, 150 mM NaCl, 10 mM EDTA for 4 days with continual orbital shaking. In replicating the work of Makarava *et al*. [12], the methodology was followed as described with the substitution of the original hamster PrP for an equivalent murine construct, PrP^23–231^. Similarly, the production of PMCA products described by Kim *et al.* [13] was carried out verbatim with the substitution of hamster PrP constructs for murine PrP^91–231^ and PrP^23–231^ as required and PMCA ’seeds' of PrP^Sc^ from RML-infected CD-1 mouse brain. In attempting to reproduce the findings of Ma and co-workers [14], we subjected murine PrP^23–231^ to the conditions described using 5% (w/v) brain homogenate from normal CD-1 mice.

### 4.8. Transmission studies in mice

Samples containing recombinant mouse PrP (residues 23–231 or 91–231) or vehicle controls were diluted in sterile Dulbecco's phosphate-buffered saline lacking calcium or magnesium ions and passed through a 25-gauge needle. CD1 mice were inoculated intracerebrally with 30 µl of solution containing between 15 and 0.015 µg of PrP. Thereafter, mice were examined daily and killed if exhibiting signs of distress or once a diagnosis of clinical prion disease was established [37]. At post-mortem brains from inoculated mice were removed and divided sagittally, with half frozen and half fixed in 10% formol-saline buffer. Clinical diagnosis of prion disease can be confounded by non-specific conditions that develop in mice as they age. We limited these confounding effects by electively culling mice after post-inoculation periods of more than 600 days and confirming clinical prion disease though neuropathological and immunohistochemical analyses. Mice were only scored positive if abnormal PrP was demonstrated in brain.

### 4.9. Neuropathological and immunohistochemical analyses of mouse brain

Fixed mouse brain was immersed in 98% formic acid for 1 h and paraffin wax embedded. Serial sections (4 µm thick) were pre-treated by boiling for 10 min in a low-ionic-strength buffer (2.1 mM Tris, 1.3 mM EDTA, 1.1 mM sodium citrate, pH 7.8) before exposure to 98% formic acid for 5 min. Abnormal PrP accumulation was examined using anti-PrP monoclonal antibody ICSM 35 (D-Gen Ltd, London) on an automated IHC staining machine (Ventana Medical Systems Inc., Tucson, Arizona) using proprietary secondary detection reagents (Ventana Medical Systems Inc) before development with 3′3 diaminobenzedine tetrachloride as the chromogen [38]. Conventional methods were used for Harris haematoxylin and eosin staining. Appropriate positive and negative controls were used throughout. Photographs were taken on an ImageView digital camera and composed with Adobe Photoshop.

## Supplementary Material

Supplementary Information

## Data accessibility

Extended data are provided in the electronic supplementary material (tables S1 and S2 and figure S1).
