# Supplementary material for: A systematic investigation of production of synthetic prions from recombinant prion protein
Source: Open Biol. 2015 Dec 2;5(12):150165. doi: 10.1098/rsob.150165 (PMC4703057; doi:10.1098/rsob.150165)
Supplement: Supplementary Information [file rsob150165supp1.pdf]

Supplementary Material for:

A systematic investigation of production of synthetic prions from recombinant prion protein

Christian Schmidt†, Jeremie Fizet†, Francesca Properzi†, Mark Batchelor, Malin K Sandberg, Julie A Edgeworth, Louise Afran, Sammy Ho, Anjna Badhan, Steffi Klier, Jacqueline M Linehan, Sebastian Brandner, Laszlo LP Hosszu, M Howard Tattum, Parmjit Jat, Anthony R Clarke, Peter C Klöhn, Jonathan DF Wadsworth, Graham S Jackson, John Collinge*

MRC Prion Unit and Department of Neurodegenerative Disease, UCL Institute of Neurology, National Hospital for Neurology and Neurosurgery, Queen Square, London WC1N 3BG, UK

†These authors contributed equally to this work.

*Correspondence should be addressed to J.C. (j.collinge@prion.ucl.ac.uk)

This pdf file contains

Supplementary Tables 1 and 2

Supplementary Figure S1

Supplementary references

Supplementary Table 1. Matrix conditions that generated positive wells according to defined criteria from recPrP*

Matrix#	Condition	Plate °Condition	NaCL mM	PrP	µg/ml	pH	DTT mM
1	Core	4°C Im	150	β	10	3.5	1
1	Core	4°C Im	150	β	10	5.5	1
1	Core	4°C O/N	0	β	10	5.5	1
1	Core	4°C O/N	150	α	1	3.5	30
1	Core	RT Im	0	α	1	5.5	0
1	Core	RT Im	0	α	1	5.5	1
1	Core	RT Im	0	α	1	5.5	30
1	Core	55°C Im	150	α	10	3.5	30
Matrix#	Condition	Plate °Condition	NaCL mM	PrP	µg/ml	pH	DTT mM
2	Thermolysin digestion	4°C Im	0	α	10	3.5	1
2	Thermolysin digestion	4°C Im	0	α	1	5.5	30
2	Thermolysin digestion	RT Im	150	α	10	3.5	0
2	Thermolysin digestion	RT O/N	0	β	10	3.5	0
2	Thermolysin digestion	RT O/N	0	β	10	3.5	0
2	Thermolysin digestion	RT O/N	150	α	10	7.5	1
Matrix#	Condition	Plate °Condition	NaCL mM	PrP	µg/ml	pH	DTT mM
4	CD1 mouse brain	RT Im	0	α	1	3.5	0
4	CD1 mouse brain	RT Im	0	α	10	3.5	0
4	CD1 mouse brain	37°C Im	0	α	1	7.5	30
4	CD1 mouse brain	55°C Im	0	α	1	5.5	30
Matrix#	Condition	Plate °Condition	NaCL mM	PrP	µg/ml	pH	DTT mM
5	Polypropylene	4°C Im	0	α	10	5.5	1
5	Polypropylene	55°C Im	0	α	10	5.5	1
5	Polypropylene	55°C Im	0	α	10	5.5	1
5	Polypropylene	55°C Im	0	β	1	3.5	1
5	Polypropylene	55°C Im	0	β	1	3.5	1
5	Polypropylene	55°C Im	150	β	1	3.5	0
5	Polypropylene	4°C O/N	0	α	1	3.5	30
5	Polypropylene	37°C O/N	0	α	1	5.5	1
Matrix#	Condition	Plate °Condition	NaCL mM	PrP	µg/ml	pH	DTT mM
6	PTFE	4°C O/N	0	α	10	5.5	1
6	PTFE	4°C O/N	0	β	1	3.5	0
6	PTFE	4°C O/N	0	β	1	3.5	1
6	PTFE	4°C O/N	150	β	1	3.5	1
Matrix#	Condition	Plate °Condition	NaCL mM	PrP	µg/ml	pH	DTT mM
8	Glass coated plate	RT Im	0	α	10	5.5	1
8	Glass coated plate	55°C Im	0	β	1	3.5	0
8	Glass coated plate	55°C O/N	150	β	1	7.5	1
Matrix#	Condition	Plate °Condition	NaCL mM	PrP	µg/ml	pH	DTT mM
11	Polypropylene	4°C Im	150	α	10	3.5	1
11	Polypropylene	37°C Im	0	α	1	5.5	1
11	Polypropylene	RT O/N	0	α	1	7.5	0
11	Polypropylene	37°C O/N	0	α	10	3.5	30
11	Polypropylene	37°C O/N	150	β	10	3.5	0
11	Polypropylene	37°C O/N	150	β	1	3.5	0

Matrix#	Condition	Plate °Condition	NaCL mM	PrP	µg/ml	pH	DTT mM
12	Glycogen	4°C Im	0	β	1	5.5	1
12	Glycogen	RT Im	0	α	1	5.5	1
12	Glycogen	RT Im	0	α	10	5.5	30
12	Glycogen	37°C Im	0	α	10	7.5	1
12	Glycogen	37°C Im	150	α	10	5.5	1
12	Glycogen	RT O/N	0	α	10	5.5	10
12	Glycogen	RT O/N	0	α	10	3.5	10
12	Glycogen	RT O/N	0	β	1	5.5	1
12	Glycogen	37°C O/N	150	α	1	3.5	1
12	Glycogen	55°C O/N	0	α	10	5.5	0
12	Glycogen	55°C O/N	0	α	10	7.5	0

Matrix#	Condition	Plate °Condition	NaCL mM	PrP	µg/ml	pH	DTT mM
13	Steel discs	4°C Im	0	β	1	3.5	1
13	Steel discs	RT Im	150	α	1	7.5	1
13	Steel discs	4°C O/N	150	α	10	7.5	1
13	Steel discs	4°C O/N	150	β	1	5.5	1
13	Steel discs	37°C O/N	150	α	1	5.5	1

Matrix#	Condition	Plate °Condition	NaCL mM	PrP	µg/ml	pH	DTT mM
15	Heparin	4°C Im	150	β	1	7.5	0
15	Heparin	37°C Im	150	β	1	3.5	0
15	Heparin	37°C Im	150	β	10	3.5	0
15	Heparin	55°C Im	0	α	10	5.5	1
15	Heparin	55°C Im	0	α	10	5.5	30
15	Heparin	55°C Im	0	α	10	7.5	30
15	Heparin	55°C Im	0	β	1	5.5	0
15	Heparin	4°C O/N	150	α	10	3.5	0
15	Heparin	RT O/N	0	β	1	7.5	0
15	Heparin	RT O/N	0	β	1	7.5	30
15	Heparin	RT O/N	150	α	1	7.5	0
15	Heparin	RT O/N	150	α	10	5.5	1
15	Heparin	RT O/N	150	β	1	3.5	0
15	Heparin	37°C O/N	150	α	1	7.5	0
15	Heparin	37°C O/N	150	α	10	3.5	0
15	Heparin	37°C O/N	150	α	10	7.5	30
15	Heparin	37°C O/N	150	β	1	3.5	30
15	Heparin	37°C O/N	150	β	1	7.5	1
15	Heparin	37°C O/N	150	β	10	3.5	0
15	Heparin	37°C O/N	150	β	10	7.5	1

Matrix#	Condition	Plate °Condition	NaCL mM	PrP	µg/ml	pH	DTT mM
16	Glycogen	4°C Im	0	α	1	5.5	0
16	Glycogen	4°C Im	0	α	10	3.5	1
16	Glycogen	4°C Im	0	β	1	7.5	0
16	Glycogen	RT Im	150	α	1	7.5	30
16	Glycogen	37°C Im	150	α	10	5.5	1
16	Glycogen	4°C O/N	0	α	1	7.5	0
16	Glycogen	4°C O/N	0	α	1	7.5	1
16	Glycogen	4°C O/N	0	α	10	3.5	30
16	Glycogen	4°C O/N	0	β	1	3.5	0
16	Glycogen	4°C O/N	0	β	1	3.5	1
16	Glycogen	4°C O/N	0	β	1	5.5	0
16	Glycogen	4°C O/N	0	β	1	7.5	0
16	Glycogen	4°C O/N	0	β	1	7.5	1
16	Glycogen	4°C O/N	0	β	10	3.5	0
16	Glycogen	4°C O/N	150	α	1	3.5	0
16	Glycogen	4°C O/N	150	α	1	3.5	30
16	Glycogen	4°C O/N	150	α	1	5.5	0
16	Glycogen	4°C O/N	150	α	1	5.5	1
16	Glycogen	4°C O/N	150	α	1	5.5	1
16	Glycogen	4°C O/N	150	α	1	7.5	0
16	Glycogen	4°C O/N	150	α	1	7.5	0
16	Glycogen	4°C O/N	150	α	1	7.5	1
16	Glycogen	4°C O/N	150	α	10	3.5	0
16	Glycogen	4°C O/N	150	α	10	3.5	0
16	Glycogen	4°C O/N	150	α	10	5.5	0

16	Glycogen	4ºC O/N	150	β	1	5.5	1
16	Glycogen	4ºC O/N	150	β	1	7.5	0
16	Glycogen	4ºC O/N	150	β	10	3.5	0
16	Glycogen	4ºC O/N	150	β	10	3.5	0
16	Glycogen	4ºC O/N	150	β	10	5.5	0
16	Glycogen	4ºC O/N	150	β	10	5.5	1
16	Glycogen	RT O/N	150	α	1	3.5	0
16	Glycogen	RT O/N	150	α	1	3.5	1
16	Glycogen	RT O/N	150	α	1	3.5	1
16	Glycogen	RT O/N	150	α	1	5.5	30
16	Glycogen	37ºC O/N	0	α	1	5.5	0
16	Glycogen	37ºC O/N	0	α	1	5.5	0
16	Glycogen	37ºC O/N	0	α	1	5.5	30
16	Glycogen	37ºC O/N	0	α	1	5.5	30
16	Glycogen	37ºC O/N	0	α	1	7.5	0
16	Glycogen	37ºC O/N	0	α	1	7.5	1
16	Glycogen	37ºC O/N	0	β	10	7.5	0
16	Glycogen	37ºC O/N	150	α	1	7.5	0
16	Glycogen	37ºC O/N	150	β	1	7.5	0
16	Glycogen	37ºC O/N	150	β	10	3.5	1
16	Glycogen	37ºC O/N	150	β	10	7.5	1

Matrix#	Condition	Plate ºCondition	NaCL mM	PrP	µg/ml	pH	DTT mM
17	Steel	55ºC Im	150	α	1	7.5	1

Matrix#	Condition	Plate ºCondition	NaCL mM	PrP	µg/ml	pH	DTT mM
18	Heparin	4ºC Im	0	α	1	5.5	30
18	Heparin	4ºC Im	150	α	1	7.5	30
18	Heparin	RT Im	0	α	10	3.5	30
18	Heparin	RT Im	150	α	10	5.5	30
18	Heparin	4ºC O/N	0	α	10	3.5	0
18	Heparin	37ºC O/N	150	β	10	7.5	0

Matrix#	Condition	Plate ºCondition	NaCL mM	PrP	µg/ml	pH	DTT mM
19	Glycogen	4ºC Im	0	α	1	3.5	1
19	Glycogen	4ºC Im	0	α	1	7.5	1
19	Glycogen	4ºC Im	150	α	1	5.5	1
19	Glycogen	RT Im	0	α	1	3.5	1
19	Glycogen	RT Im	0	α	10	5.5	30
19	Glycogen	37ºC Im	0	α	10	7.5	1
19	Glycogen	37ºC Im	0	β	1	5.5	0
19	Glycogen	37ºC Im	0	β	1	7.5	0
19	Glycogen	4ºC O/N	0	β	10	5.5	30
19	Glycogen	37ºC O/N	150	α	10	5.5	0
19	Glycogen	55ºC O/N	0	α	10	5.5	1

Matrix#	Condition	Plate ºCondition	NaCL mM	PrP	µg/ml	pH	DTT mM
20	'+/- Pro-Ject	RT Im	150	α	10	3.5	0

Matrix#	Condition	Plate ºCondition	NaCL mM	PrP	µg/ml	pH	DTT mM
21	Glycogen/Heparin	55ºC Im	150	α+Glycogen	1	7.5	0
21	Glycogen/Heparin	4ºC O/N	0	α+Glycogen	1	3.5	0
21	Glycogen/Heparin	4ºC O/N	0	α+Glycogen	1	3.5	1
21	Glycogen/Heparin	4ºC O/N	0	α+Glycogen	10	3.5	0
21	Glycogen/Heparin	4ºC O/N	0	α+Glycogen	10	5.5	0
21	Glycogen/Heparin	4ºC O/N	0	α+Glycogen	10	7.5	0
21	Glycogen/Heparin	4ºC O/N	0	α+Glycogen	10	7.5	1
21	Glycogen/Heparin	4ºC O/N	0	α+Heparin	1	5.5	0
21	Glycogen/Heparin	4ºC O/N	0	α+Heparin	10	3.5	0
21	Glycogen/Heparin	4ºC O/N	150	α+Glycogen	1	7.5	0
21	Glycogen/Heparin	RT O/N	0	α+Glycogen	1	7.5	1
21	Glycogen/Heparin	RT O/N	150	α+Glycogen	1	3.5	0
21	Glycogen/Heparin	37ºC O/N	0	α+Glycogen	10	3.5	0
21	Glycogen/Heparin	55ºC O/N	0	α+Heparin	1	3.5	1
21	Glycogen/Heparin	55ºC O/N	0	α+Glycogen	1	7.5	1
21	Glycogen/Heparin	55ºC O/N	0	α+Glycogen	1	7.5	1

21	Glycogen/Heparin	55ºC O/N	0	α+Glycogen	10	3.5	0
21	Glycogen/Heparin	55ºC O/N	0	α+Glycogen	10	5.5	30
21	Glycogen/Heparin	55ºC O/N	0	α+Heparin	1	3.5	0
21	Glycogen/Heparin	55ºC O/N	0	α+Heparin	1	3.5	1
21	Glycogen/Heparin	55ºC O/N	0	α+Heparin	1	5.5	0
21	Glycogen/Heparin	55ºC O/N	0	α+Heparin	1	5.5	1
21	Glycogen/Heparin	55ºC O/N	0	α+Heparin	1	7.5	0
21	Glycogen/Heparin	55ºC O/N	0	α+Heparin	1	7.5	30
21	Glycogen/Heparin	55ºC O/N	0	α+Heparin	10	3.5	0
21	Glycogen/Heparin	55ºC O/N	0	α+Heparin	10	3.5	30
21	Glycogen/Heparin	55ºC O/N	150	α+Glycogen	1	3.5	1
21	Glycogen/Heparin	55ºC O/N	150	α+Glycogen	10	3.5	0
21	Glycogen/Heparin	55ºC O/N	150	α+Glycogen	10	5.5	1
21	Glycogen/Heparin	55ºC O/N	150	α+Glycogen	10	7.5	1
21	Glycogen/Heparin	55ºC O/N	150	α+Glycogen	10	7.5	30
21	Glycogen/Heparin	55ºC O/N	150	α+Heparin	1	3.5	0
21	Glycogen/Heparin	55ºC O/N	150	α+Heparin	1	3.5	1
21	Glycogen/Heparin	55ºC O/N	150	α+Heparin	1	3.5	30
21	Glycogen/Heparin	55ºC O/N	150	α+Heparin	1	7.5	0
21	Glycogen/Heparin	55ºC O/N	150	α+Heparin	10	3.5	0
21	Glycogen/Heparin	55ºC O/N	150	α+Heparin	10	3.5	1
21	Glycogen/Heparin	55ºC O/N	150	α+Heparin	10	5.5	0
21	Glycogen/Heparin	55ºC O/N	150	α+Heparin	10	7.5	1

Matrix#	Condition	Plate ºCondition	NaCL mM	PrP	µg/ml	pH	DTT mM
22	Brain	4ºC Im	0	α	1	7.5	1
22	Brain	4ºC Im	0	α	1	7.5	30
22	Brain	4ºC Im	0	α	10	3.5	30
22	Brain	RT Im	150	α	1	7.5	1
22	Brain	RT Im	150	α	1	7.5	30
22	Brain	RT Im	150	α	10	3.5	1
22	Brain	RT Im	150	α	10	3.5	1
22	Brain	RT Im	150	α	10	3.5	30
22	Brain	RT Im	150	α	10	3.5	30
22	Brain	RT Im	150	α	10	5.5	1
22	Brain	RT Im	150	α	10	5.5	30
22	Brain	RT Im	150	α	10	5.5	30
22	Brain	RT Im	150	β	1	3.5	1
22	Brain	RT Im	150	β	1	3.5	30
22	Brain	37ºC Im	0	α	10	3.5	1
22	Brain	37ºC Im	0	α	10	3.5	1
22	Brain	37ºC Im	0	α	10	3.5	30
22	Brain	37ºC Im	0	α	10	3.5	30
22	Brain	37ºC Im	0	α	10	5.5	1
22	Brain	37ºC Im	0	α	10	5.5	1
22	Brain	37ºC Im	0	α	10	5.5	30
22	Brain	37ºC Im	0	α	10	5.5	30
22	Brain	37ºC Im	0	α	10	7.5	30
22	Brain	55ºC Im	0	α	10	3.5	1
22	Brain	55ºC Im	150	α	1	5.5	1
22	Brain	55ºC Im	150	α	1	7.5	1
22	Brain	55ºC Im	150	α	1	7.5	30
22	Brain	55ºC Im	150	α	10	3.5	30
22	Brain	55ºC Im	150	α	10	5.5	30
22	Brain	55ºC Im	150	α	10	5.5	30
22	Brain	4ºC O/N	0	α	1	5.5	30

22	Brain	4ºC O/N	0	α	10	3.5	1
22	Brain	4ºC O/N	0	α	10	3.5	30
22	Brain	4ºC O/N	0	α	10	5.5	1
22	Brain	4ºC O/N	0	α	10	7.5	30
22	Brain	4ºC O/N	150	α	10	3.5	0
22	Brain	RT O/N	0	α	1	5.5	30
22	Brain	RT O/N	0	α	10	5.5	30
22	Brain	55ºC O/N	0	α	10	3.5	0
22	Brain	55ºC O/N	0	α	10	3.5	1
22	Brain	55ºC O/N	0	α	10	5.5	30
22	Brain	55ºC O/N	0	α	10	7.5	30

Matrix#	Condition	Plate ºCondition	NaCL mM	PrP	µg/ml	pH	DTT mM
21b	Glycogen/Heparin	RT Im	0	α+Glycogen	10	7.5	0
21b	Glycogen/Heparin	55ºC Im	150	α+Glycogen	10	3.5	1
21b	Glycogen/Heparin	55ºC Im	150	α+Glycogen	10	5.5	1
21b	Glycogen/Heparin	4ºC O/N	0	α+Heparin	1	5.5	1
21b	Glycogen/Heparin	55ºC O/N	0	α+Glycogen	1	3.5	1
21b	Glycogen/Heparin	55ºC O/N	0	α+Glycogen	1	3.5	1
21b	Glycogen/Heparin	55ºC O/N	0	α+Glycogen	1	7.5	1
21b	Glycogen/Heparin	55ºC O/N	0	α+Glycogen	10	5.5	1
21b	Glycogen/Heparin	55ºC O/N	0	α+Glycogen	10	7.5	1

Matrix#	Condition	Plate ºCondition	NaCL mM	PrP	µg/ml	pH	DTT mM
24	Metals (1:1Cu)	4ºC O/N	150	α	10	5.5	30

Matrix#	Condition	Plate ºCondition	NaCL mM	PrP	µg/ml	pH	DTT mM
25	5:1 Cu	4ºC Im	0	α	1	7.5	0
25	5:1 Cu	4ºC Im	0	α	10	5.5	1
25	5:1 Cu	4ºC Im	0	α	10	5.5	1
25	5:1 Cu	4ºC Im	0	β	1	3.5	1
25	5:1 Cu	4ºC Im	150	α	1	7.5	30
25	5:1 Cu	RT Im	150	α	10	3.5	0
25	5:1 Cu	RT Im	150	α	10	5.5	0
25	5:1 Cu	37ºC Im	0	α	1	5.5	30
25	5:1 Cu	37ºC Im	0	α	1	7.5	30
25	5:1 Cu	37ºC Im	0	α	10	5.5	1
25	5:1 Cu	37ºC Im	0	β	1	7.5	0
25	5:1 Cu	37ºC Im	0	β	10	5.5	0
25	5:1 Cu	55ºC Im	150	α	10	5.5	1
25	5:1 Cu	55ºC Im	150	β	10	5.5	1
25	5:1 Cu	55ºC Im	150	β	10	5.5	30
25	5:1 Cu	4ºC O/N	0	α	1	7.5	30
25	5:1 Cu	4ºC O/N	0	α	10	3.5	30
25	5:1 Cu	4ºC O/N	0	α	10	5.5	30
25	5:1 Cu	4ºC O/N	0	α	10	5.5	30
25	5:1 Cu	4ºC O/N	0	α	10	7.5	10
25	5:1 Cu	4ºC O/N	0	α	10	7.5	10
25	5:1 Cu	4ºC O/N	0	β	10	3.5	0
25	5:1 Cu	4ºC O/N	0	β	10	3.5	0
25	5:1 Cu	4ºC O/N	0	β	10	3.5	1
25	5:1 Cu	4ºC O/N	0	β	10	3.5	1
25	5:1 Cu	4ºC O/N	0	β	10	5.5	0
25	5:1 Cu	4ºC O/N	0	β	10	5.5	1
25	5:1 Cu	4ºC O/N	0	β	10	5.5	1
25	5:1 Cu	4ºC O/N	0	β	10	7.5	0
25	5:1 Cu	4ºC O/N	0	β	10	7.5	0
25	5:1 Cu	4ºC O/N	0	β	10	7.5	1
25	5:1 Cu	4ºC O/N	150	α	1	7.5	0
25	5:1 Cu	37ºC O/N	150	α	1	3.5	1
25	5:1 Cu	37ºC O/N	150	α	1	7.5	0
25	5:1 Cu	37ºC O/N	150	α	10	3.5	1
25	5:1 Cu	37ºC O/N	150	α	10	3.5	1
25	5:1 Cu	37ºC O/N	150	α	10	3.5	10
25	5:1 Cu	37ºC O/N	150	α	10	7.5	0
25	5:1 Cu	37ºC O/N	150	α	10	7.5	10
25	5:1 Cu	37ºC O/N	150	β	1	3.5	0
25	5:1 Cu	37ºC O/N	150	β	1	5.5	0
25	5:1 Cu	37ºC O/N	150	β	1	5.5	1

25	5:1 Cu	37ºC O/N	150	β	1	7.5	0
25	5:1 Cu	37ºC O/N	150	β	1	7.5	1
25	5:1 Cu	37ºC O/N	150	β	10	3.5	0
25	5:1 Cu	37ºC O/N	150	β	10	3.5	1
25	5:1 Cu	55ºC O/N	0	α	1	7.5	1
25	5:1 Cu	55ºC O/N	0	α	10	7.5	1
25	5:1 Cu	55ºC O/N	0	β	1	3.5	1
25	5:1 Cu	55ºC O/N	0	β	1	7.5	1

Matrix#	Condition	Plate ºCondition	NaCL mM	PrP	µg/ml	pH	DTT mM
26	5:1 Zn	4ºC Im	0	β	10	3.5	30
26	5:1 Zn	37ºC Im	150	β	10	5.5	0
26	5:1 Zn	55ºC Im	0	β	10	5.5	0

Matrix#	Condition	Plate ºCondition	NaCL mM	PrP	µg/ml	pH	DTT mM
26b	5:1 Zn	4ºC Im	0	α	1	7.5	1
26b	5:1 Zn	4ºC Im	0	α	1	7.5	30
26b	5:1 Zn	4ºC Im	0	α	10	3.5	30
26b	5:1 Zn	RT	150	α	1	7.5	1
26b	5:1 Zn	RT	150	α	1	7.5	30
26b	5:1 Zn	RT	150	α	10	3.5	1
26b	5:1 Zn	RT	150	α	10	3.5	1
26b	5:1 Zn	RT	150	α	10	3.5	30
26b	5:1 Zn	RT	150	α	10	3.5	30
26b	5:1 Zn	RT	150	α	10	5.5	1
26b	5:1 Zn	RT	150	α	10	5.5	30
26b	5:1 Zn	RT	150	α	10	5.5	30
26b	5:1 Zn	RT	150	α	1	3.5	1
26b	5:1 Zn	RT	150	α	1	3.5	30
26b	5:1 Zn	37ºC Im	0	α	10	3.5	1
26b	5:1 Zn	37ºC Im	0	α	10	3.5	1
26b	5:1 Zn	37ºC Im	0	α	10	3.5	30
26b	5:1 Zn	37ºC Im	0	α	10	3.5	30
26b	5:1 Zn	37ºC Im	0	α	10	5.5	1
26b	5:1 Zn	37ºC Im	0	α	10	5.5	1
26b	5:1 Zn	37ºC Im	0	α	10	5.5	30
26b	5:1 Zn	37ºC Im	0	α	10	5.5	30
26b	5:1 Zn	37ºC Im	0	α	10	7.5	30
26b	5:1 Zn	55ºC Im	0	α	1	3.5	1
26b	5:1 Zn	55ºC Im	150	α	1	5.5	1
26b	5:1 Zn	55ºC Im	150	α	1	7.5	1
26b	5:1 Zn	55ºC Im	150	α	1	7.5	10
26b	5:1 Zn	55ºC Im	150	α	10	3.5	10
26b	5:1 Zn	55ºC Im	150	α	10	5.5	10
26b	5:1 Zn	55ºC Im	150	α	10	5.5	10

Matrix#	Condition	Plate ºCondition	NaCL mM	PrP	µg/ml	pH	DTT mM
25b	Brain homogenate + 5:1 Cu	4ºC Im	0	α	1	7.5	0
25b	Brain homogenate + 5:1 Cu	4ºC Im	0	α	10	5.5	1
25b	Brain homogenate + 5:1 Cu	4ºC Im	0	α	10	5.5	1
25b	Brain homogenate + 5:1 Cu	4ºC Im	0	β	1	3.5	1
25b	Brain homogenate + 5:1 Cu	4ºC Im	150	α	1	7.5	30
25b	Brain homogenate + 5:1 Cu	RT Im	150	α	10	3.5	0
25b	Brain homogenate + 5:1 Cu	RT Im	150	α	10	5.5	0
25b	Brain homogenate + 5:1 Cu	37ºC Im	0	α	1	5.5	30
25b	Brain homogenate + 5:1 Cu	37ºC Im	0	α	1	7.5	30
25b	Brain homogenate + 5:1 Cu	37ºC Im	0	α	10	5.5	1
25b	Brain homogenate + 5:1 Cu	37ºC Im	0	β	1	7.5	0
25b	Brain homogenate + 5:1 Cu	37ºC Im	0	β	10	5.5	0
25b	Brain homogenate + 5:1 Cu	55ºC Im	150	α	10	5.5	1
25b	Brain homogenate + 5:1 Cu	55ºC Im	150	β	10	5.5	1
25b	Brain homogenate + 5:1 Cu	55ºC Im	150	β	10	5.5	10
25b	Brain homogenate + 5:1 Cu	4ºC O/N	0	α	1	7.5	30
25b	Brain homogenate + 5:1 Cu	4ºC O/N	0	α	10	3.5	30
25b	Brain homogenate + 5:1 Cu	4ºC O/N	0	α	10	5.5	30
25b	Brain homogenate + 5:1 Cu	4ºC O/N	0	α	10	5.5	30
25b	Brain homogenate + 5:1 Cu	4ºC O/N	0	α	10	7.5	1
25b	Brain homogenate + 5:1 Cu	4ºC O/N	0	α	10	7.5	1

25b	Brain homogenate + 5:1 Cu	4°C O/N	0	β	10	3.5	0
25b	Brain homogenate + 5:1 Cu	4°C O/N	0	β	10	3.5	0
25b	Brain homogenate + 5:1 Cu	4°C O/N	0	β	10	3.5	1
25b	Brain homogenate + 5:1 Cu	4°C O/N	0	β	10	3.5	1
25b	Brain homogenate + 5:1 Cu	4°C O/N	0	β	10	5.5	0
25b	Brain homogenate + 5:1 Cu	4°C O/N	0	β	10	5.5	1
25b	Brain homogenate + 5:1 Cu	4°C O/N	0	β	10	5.5	1
25b	Brain homogenate + 5:1 Cu	4°C O/N	0	β	10	7.5	0
25b	Brain homogenate + 5:1 Cu	4°C O/N	0	β	10	7.5	0
25b	Brain homogenate + 5:1 Cu	4°C O/N	0	β	10	7.5	1
25b	Brain homogenate + 5:1 Cu	4°C O/N	150	α	1	7.5	0

Matrix#	Condition	Plate °Condition	NaCL mM	PrP	µg/ml	pH	DTT mM
25c	5:1 Cu	4°C Im	0	α	1	3.5	0
25c	5:1 Cu	4°C Im	0	α	1	3.5	0
25c	5:1 Cu	4°C Im	0	α	1	7.5	0
25c	5:1 Cu	4°C Im	0	α	1	7.5	30
25c	5:1 Cu	4°C Im	0	β	10	3.5	0
25c	5:1 Cu	4°C Im	150	α	1	3.5	1
25c	5:1 Cu	4°C Im	150	α	1	5.5	0
25c	5:1 Cu	4°C Im	150	α	10	3.5	0
25c	5:1 Cu	4°C Im	150	α	10	5.5	0
25c	5:1 Cu	4°C Im	150	β	10	5.5	0
25c	5:1 Cu	RT Im	0	α	1	7.5	0
25c	5:1 Cu	RT Im	0	α	10	3.5	0
25c	5:1 Cu	RT Im	150	β	1	5.5	0
25c	5:1 Cu	37°C Im	150	α	1	7.5	0
25c	5:1 Cu	55°C Im	0	α	1	7.5	0
25c	5:1 Cu	55°C Im	150	β	1	5.5	0
25c	5:1 Cu	4°C O/N	150	β	10	5.5	0
25c	5:1 Cu	RT O/N	0	α	10	3.5	0
25c	5:1 Cu	RT O/N	150	α	1	5.5	0
25c	5:1 Cu	RT O/N	150	α	1	7.5	1
25c	5:1 Cu	37°C O/N	0	β	1	5.5	0
25c	5:1 Cu	37°C O/N	0	β	10	7.5	0
25c	5:1 Cu	37°C O/N	150	α	1	7.5	1
25c	5:1 Cu	55°C O/N	0	α	1	3.5	30
25c	5:1 Cu	55°C O/N	0	α	1	7.5	30
25c	5:1 Cu	55°C O/N	150	α	10	5.5	0
25c	5:1 Cu	55°C O/N	150	α	10	7.5	0

Matrix#	Condition	Plate °Condition	NaCL mM	PrP	µg/ml	pH	DTT mM
25d	5:1 Cu	55°C Im	0	α	10	7.5	10
25d	5:1 Cu	55°C Im	150	α	10	7.5	100
25d	5:1 Cu	4°C O/N	150	α	100	3.5	10
25d	5:1 Cu	RT O/N	0	α	100	3.5	100
25d	5:1 Cu	37 O/N	0	α	10	5.5	10
25d	5:1 Cu	37 O/N	0	α	10	7.5	10

Matrix#	Condition	Plate °Condition	NaCL mM	PrP	µg/ml	pH	DTT mM
25e	5:1 Cu	RT Im	0	α	100	3.5	10
25e	5:1 Cu	55°C Im	150	α	10	3.5	100
25e	5:1 Cu	4°C O/N	150	α	10	3.5	10
25e	5:1 Cu	RT O/N	0	α	100	7.5	10
25e	5:1 Cu	RT O/N	150	α	10	5.5	10
25e	5:1 Cu	RT O/N	150	α	100	5.5	10
25e	5:1 Cu	55 O/N	0	α	100	7.5	100
25e	5:1 Cu	55 O/N	0	α	100	7.5	100
25e	5:1 Cu	RT Im	150	α	10	5.5	10

Matrix#	Condition	Plate °Condition	NaCL mM	ThioFlαvin	RML	EDTA	PolyA
Fibrils	Fibrils	4°C Im	0	n	y	n	y
Fibrils	Fibrils	4°C Im	0	n	y	n	y
Fibrils	Fibrils	4°C Im	0	n	y	n	y
Fibrils	Fibrils	4°C Im	0	n	n	n	y
Fibrils	Fibrils	4°C Im	0	n	n	y	n
Fibrils	Fibrils	4°C Im	0	y	y	y	y
Fibrils	Fibrils	4°C Im	0	y	n	n	y
Fibrils	Fibrils	4°C Im	150	n	y	y	y
Fibrils	Fibrils	4°C Im	150	n	y	y	y
Fibrils	Fibrils	4°C Im	150	n	y	y	y
Fibrils	Fibrils	4°C Im	150	n	n	n	y
Fibrils	Fibrils	4°C Im	150	y	y	n	y
Fibrils	Fibrils	4°C Im	150	y	y	y	y
Fibrils	Fibrils	4°C Im	150	y	n	y	n
Fibrils	Fibrils	RT Im	0	n	y	y	y
Fibrils	Fibrils	RT Im	0	n	y	y	n
Fibrils	Fibrils	RT Im	0	n	y	n	n
Fibrils	Fibrils	RT Im	0	n	y	y	y
Fibrils	Fibrils	RT Im	0	n	y	n	y
Fibrils	Fibrils	RT Im	0	n	n	n	y
Fibrils	Fibrils	RT Im	0	y	y	n	y
Fibrils	Fibrils	RT Im	0	y	y	n	n
Fibrils	Fibrils	RT Im	0	y	y	y	n
Fibrils	Fibrils	RT Im	0	y	n	n	n
Fibrils	Fibrils	RT Im	150	n	y	n	y
Fibrils	Fibrils	RT Im	150	n	y	y	y
Fibrils	Fibrils	RT Im	150	n	n	n	y
Fibrils	Fibrils	RT Im	150	n	n	n	n
Fibrils	Fibrils	RT Im	150	n	n	n	y
Fibrils	Fibrils	RT Im	150	n	n	n	n
Fibrils	Fibrils	RT Im	150	y	y	y	n
Fibrils	Fibrils	RT Im	150	y	y	n	n
Fibrils	Fibrils	RT Im	150	y	y	n	y
Fibrils	Fibrils	RT Im	150	y	y	y	n
Fibrils	Fibrils	RT Im	150	y	y	n	y
Fibrils	Fibrils	RT Im	150	y	y	n	n
Fibrils	Fibrils	RT Im	150	y	n	y	n

Matrix#	Condition	Plate °Condition	NaCL mM	PrP	μg/ml	pH	DTT mM
25f	5:1 Cu	RT Im	150	α	10	3.5	100
25f	5:1 Cu	RT Im	150	α	10	5.5	100
25f	5:1 Cu	55°C Im	0	α	10	3.5	10
25f	5:1 Cu	55°C Im	150	α	10	7.5	10
25f	5:1 Cu	37°C O/N	0	α	10	3.5	10
25f	5:1 Cu	55°C O/N	150	α	10	5.5	10

Matrix#	Condition	Plate °Condition	NaCL mM	PrP	μg/ml	pH	DTT mM
25 g	5:1 Cu	4°C Im	0	β	10	7.5	0
25 g	5:1 Cu	4°C Im	0	β	10	7.5	0
25 g	5:1 Cu	4°C Im	0	β	10	7.5	0
25 g	5:1 Cu	4°C Im	0	β	10	7.5	0
25 g	5:1 Cu	4°C Im	0	β	10	7.5	0
25 g	5:1 Cu	4°C Im	0	β	10	7.5	0
25 g	5:1 Cu	4°C Im	0	β	10	7.5	0
25 g	5:1 Cu	4°C Im	0	β	10	7.5	0
25 g	5:1 Cu	4°C Im	0	β	10	7.5	0
25 g	5:1 Cu	4°C Im	0	β	10	7.5	0

Matrix#	Condition	Plate °Condition	NaCL mM	PrP	μg/ml	pH	DTT mM
25i	5:1 Cu	RT O/N	0	β	1	5.5	30

Matrix#	Condition	Plate °Condition	NaCL mM	Cu	DTT mM	pH	Glycine mM
02 Matrix 2	PolyA	4°C Im	0	1:10	1	5.5	0
02 Matrix 2	PolyA	4°C Im	0	1:5	0	3.5	1
02 Matrix 2	PolyA	RT Im	0	1:10	0	5.5	0
02 Matrix 2	PolyA	RT Im	150	1:1	0	7.5	0
02 Matrix 2	PolyA	RT Im	0	1:5	1	3.5	0
02 Matrix 2	PolyA	4°C O/N	0	1:5	1	7.5	0
02 Matrix 2	PolyA	4°C O/N	0	1:1	0	5.5	1

Matrix#	Condition	Plate °Condition	NaCL mM	Cu	DTT mM	pH	Glycine mM
04 Matrix 2	PolyA (Investig. of Matrix2_02 & 03)	55°C Im no Re°CPrP	0	1:10	0	5.5	0
04 Matrix 2	PolyA (Investig. of Matrix2_02 & 03)	55°C Im +Re°CPrP DoubleKD N2A	0	1:5	0	3.5	1
04 Matrix 2	PolyA (Investig. of Matrix2_02 & 03)	°Core°Conditions	0	1:5	0	5.5	0

Matrix#	Condition	Plate °Condition	NaCL mM	Cu	DTT mM	pH	Glycine mM
05 Matrix 2	PolyA (Investig. of Matrix2_02 & 03 & 04)	55°C Im+Re°CPrP N2A DKD	150	1:5	1	5.5	1
05 Matrix 2	PolyA (Investig. of Matrix2_02 & 03 & 04)	55°C Im+Re°CPrP N2A DKD	0	1:1	1	7.5	1
05 Matrix 2	PolyA (Investig. of Matrix2_02 & 03 & 04)	55°C Im+RML PK1 DKD	0	1:1	1	5.5	0
05 Matrix 2	PolyA (Investig. of Matrix2_02 & 03 & 04)	55°C Im+RML PK1 DKD	150	1:5	0	5.5	1

Matrix#	Condition	Plate °Condition	NaCL mM	Cu	DTT mM	pH	Glycine mM
06 Matrix 2	POPG	4°C Im	150	1:10	1	5.5	1
06 Matrix 2	POPG	RT Im	150	1:5	0	3.5	0

Matrix#	Condition	Plate °Condition	NaCL mM	Cu	DTT mM	pH	Glycine mM
07 Matrix 2	POPE	37°C Im	0	1:10	0	5.5	1
07 Matrix 2	POPE	37°C Im	150	1:10	0	7.5	1
07 Matrix 2	POPE	4°C O/N	150	1:1	0	5.5	0
07 Matrix 2	POPE	55°C O/N	0	1:1	1	7.5	0

Matrix#	Condition	Plate °Condition	NaCL mM	Cu	DTT mM	pH	Glycine mM
316W Matrix 2	steel wires AISI 316	4°C Im	0	1:10	0	7.5	1
316W Matrix 2	steel wires AISI 316	4°C O/N	150	1:1	0	5.5	0
316W Matrix 2	steel wires AISI 316	55°C O/N	0	1:1	1	5.5	1

Matrix#	Condition	Plate °Condition	NaCL mM	Cu	DTT mM	pH	Glycine mM
304W Matrix 2	Steel wires AISI 304	37°C Im	0	1:5	1	5.5	1
304W Matrix 2	Steel wires AISI 304	55°C Im	150	1:5	0	5.5	0
304W Matrix 2	Steel wires AISI 304	55°C Im	0	1:1	0	5.5	0
304W Matrix 2	Steel wires AISI 304	RT O/N	150	1:10	1	3.5	1

Matrix#	Condition	Plate °Condition	NaCL mM	Cu	DTT mM	pH	Glycine mM
09 Matrix 2	PK1/2 Cell Membranes	4°C Im	150	1:10	0	3.5	0
09 Matrix 2	PK1/2 Cell Membranes	55°C Im	0	1:1	0	7.5	0
09 Matrix 2	PK1/2 Cell Membranes	RT O/N	0	1:1	0	7.5	0
09 Matrix 2	PK1/2 Cell Membranes	RT O/N	150	1:1	1	5.5	1

Matrix#	Condition	Plate °Condition	NaCL mM	Cu	DTT mM	pH	Glycine mM
316D Matrix 2	Steel disks AISI 316	4°C O/N	0	1:1	0	3.5	0

*The table describes the matrix core conditions and additive combinations that led to positives consistent with infected cell cultures. All of the conditions listed produced ASCA results that were scored positive on the basis of three criteria: 1) significant spot counts (>background mean + 10 standard deviations), 2) evidence of prion propagation (increasing spot counts over two successive cell splits measured between splits three and five) and 3) the reproducibility of duplicates for each sample. Despite multiple attempts, none of these conditions reproducibly generated synthetic prions. Full details can be supplied on request.

Key
Im Immediate. PrP master plates were incubated for 1 h prior to infecting cells.
O/N Overnight. PrP master plates were incubated for 16 h prior to infecting cells.

Supplementary Table 2. Mouse bioassay of recPrP samples generated using published methods for prion synthesis

Inoculum*	Published method	ASCA†	Rodent bioassay Attack rate‡
0.5 mg/ml fibrillised recombinant murine PrP^{91-231}. (Diluted 10^{-1} for inoculation of 30 μl).	Legname et al, Science, 2004. Ref N° 1	No detectable infectivity	0/15
0.1 mg/ml fibrillised recombinant murine PrP^{23-231}, annealed with 5% (w/v) normal murine (CD-1) brain homogenate. (Diluted 10^{-1} for inoculation of 30 μl).	Makarava et al, Acta Neuropathologica, 2010. Ref N° 2	No detectable infectivity	0/15
0.1 mg/ml fibrillised recombinant murine PrP^{23-231}, annealed with 5% (w/v) BSA (Fraction V). (Diluted 10^{-1} for inoculation of 30 μl).	Makarava et al, Acta Neuropathologica, 2010. Ref N° 2	No detectable infectivity	0/15
PMCA product containing 50 μg/ml recombinant murine PrP^{23-231} (30 μl inoculated).	Kim et al, Journal of Biological Chemistry, 2010. Ref N° 3	No detectable infectivity	0/15
PMCA product containing 50 μg/ml recombinant murine PrP^{91-231}. (30 μl inoculated).	Kim et al, Journal of Biological Chemistry, 2010. Ref N° 3	No detectable infectivity	0/15
PMCA product containing 500 ng/ml recombinant murine PrP^{23-231} and 5% (w/v) normal mouse (CD-1) brain homogenate. (30 μl inoculated).	Wang et al, Science, 2010. Ref N° 4	No detectable infectivity	0/15
PBS Sentinel control. (30 μl inoculated).		Not tested	0/15

* Samples containing recombinant PrP were either diluted 10-fold (in sterile Dulbecco's phosphate buffered saline lacking calcium or magnesium ions) or used undiluted and passed through a 25 gauge needle before use. Each mouse was inoculated intracerebrally with 30 µl of solution containing between 1.5 and 0.015 µg of PrP.

† Automated Scrapie Cell Assay (ASCA) measurement of prion infectivity. All samples were applied as 10 µl of original, undiluted inoculum mixed with 90 µl of fresh OFCS medium (Opti-MEM, containing 10% fetal calf serum; 100 U/ml penicillin and 100 µg/ml streptomycin; Invitrogen, UK). All samples were tested n=6 with each ASCA well containing between 5 and 0.005 µg of PrP.

‡ Attack rate in rodent bioassay is defined as the total number of animals clinically affected or sub-clinically infected with prion disease as a proportion of the total number of inoculated mice. Mice were culled after incubation periods >600 days. Presence of prion disease or subclinical prion infection was determined by a clinical signs and neuropathological examination and PrP-immunohistochemical analyses of post-mortem brain. Mice were only scored positive if abnormal PrP deposition was demonstrated in brain.

Figure S1

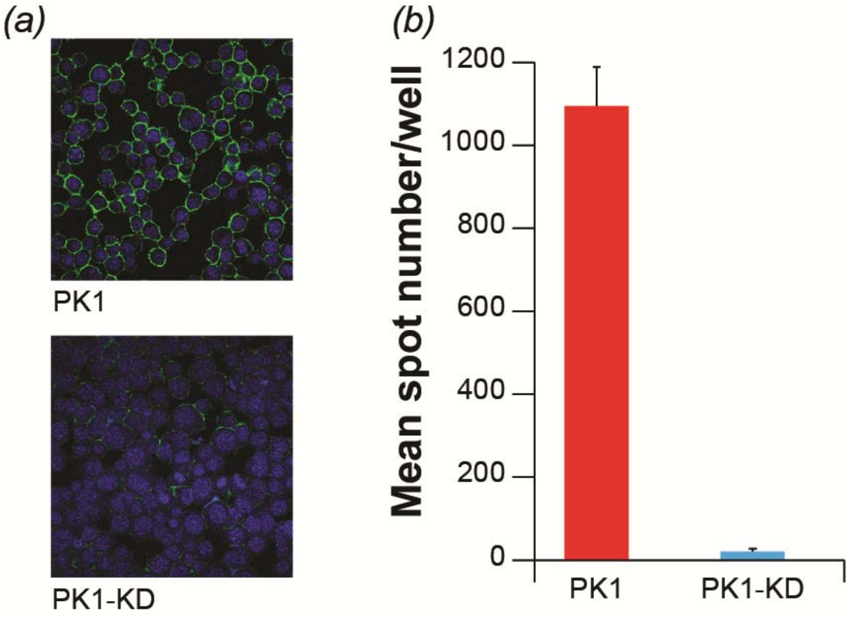

Supplementary Figure S1 Establishing a *Prnp* knockdown cell line refractory to prion infection. *Prnp* expression was silenced in susceptible PK1 cells followed by infection with a 10^{-5} dilution of 10% (w/v) RML brain homogenate to verify that cells are refractory to prion infection when measured using the ASCA. (A) Cell surface expression of PrP^C in uninfected PK1 and *Prnp*-silenced PK1-knockdown cells (PK1-KD) detected with anti-PrP antibody ICSM18. (B) The number of infected cells (mean spot number ± SEM) determined at the fourth cell split.

Supplementary References

1. Legname,G. *et al.* 2004 Synthetic mammalian prions. *Science* **305**, 673-676.

2. Makarava, N. *et al.* 2010 Recombinant prion protein induces a new transmissible prion disease in wild-type animals. *Acta Neuropathol.* **119**, 177-187.

3. Kim, J.I. *et al.* 2010 Mammalian prions generated from bacterially expressed prion protein in the absence of any mammalian cofactors. *J. Biol. Chem.* **285**, 14083-14087.

4. Wang, F., Wang, X., Yuan, C.G., & Ma, J. 2010 Generating a prion with bacterially expressed recombinant prion protein. *Science* **327**, 1132-1135.
